# Impact of a Pro-Active Infectious Disease Consultation on the Management of a Multidrug-Resistant Organisms Outbreak in a COVID-19 Hospital: A Three-Months Quasi-Experimental Study

**DOI:** 10.3390/antibiotics12040712

**Published:** 2023-04-06

**Authors:** Davide Fiore Bavaro, Nicolò De Gennaro, Alessandra Belati, Lucia Diella, Roberta Papagni, Luisa Frallonardo, Michele Camporeale, Giacomo Guido, Carmen Pellegrino, Maricla Marrone, Alessandro Dell’Erba, Loreto Gesualdo, Nicola Brienza, Salvatore Grasso, Giuseppe Columbo, Antonio Moschetta, Giovanna Elisiana Carpagnano, Antonio Daleno, Anna Maria Minicucci, Giovanni Migliore, Annalisa Saracino

**Affiliations:** 1 Clinic of Infectious Diseases, Department of Precision and Regenerative Medicine and Ionian Area, University of Bari Aldo Moro, 70124 Bari, Italy; 2 Interdisciplinary Department of Medicine, University of Bari–Section of Legal Medicine, Bari General Hospital, 70124 Bari, Italy; 3 Precision and Regenerative Medicine and Ionian Area, Nephrology, Dialysis and Transplantation Unit, University of Bari Aldo Moro, 70124 Bari, Italy; 4 Precision and Regenerative Medicine and Ionian Area, Section of Anesthesia and Intensive Care, University of Bari Aldo Moro, 70124 Bari, Italy; 5 Department of Interdisciplinary Medicine, University of Bari Aldo Moro, 70124 Bari, Italy; 6 Precision and Regenerative Medicine and Ionian Area, Neuroscience, and Sense Organs, University of Bari Aldo Moro, 70124 Bari, Italy; 7 Section of Health Management, Policlinico Hospital, 70124 Bari, Italy; 8 General Direction, Policlinico Hospital, 70124 Bari, Italy

**Keywords:** multidrug-resistant organisms, COVID-19, KPC, *Acinetobacter baumannii*, Infectious Diseases Consultation, bacterial infections

## Abstract

Background: Antimicrobial and diagnostic stewardship (AS/DS) principles are crucial for the management of multidrug-resistant organisms (MDROs) infections. We evaluated the impact of a pro-active Infectious Disease (ID) consultation on the mortality risk of patients during an MDROs outbreak in a COVID-19 hospital. Methods: A quasi-experimental study was performed in a dedicated COVID-19 hospital, including patients with suspected/confirmed infection and/or colonization by MDROs, which were managed as follows: (i) according to the standard of care during the pre-phase and (ii) in collaboration with a dedicated ID team performing a pro-active bedside evaluation every 48–72 h in the post-phase. Results: Overall, 112 patients were included (pre-phase = 89 and post-phase = 45). The AS interventions included the following: therapy optimization (33%), de-escalation to narrow the spectrum (24%) or to lessen toxic drugs (20%), and discontinuation of antimicrobials (64%). DS included the request of additional microbiologic tests (82%) and instrumental exams (16%). With the Cox model, after adjusting for age, sex, COVID-19 severity, infection source, etiological agents, and post-phase attendance, only age predicted an increased risk of mortality, while attendance in the post-phase resulted in a decreased risk of mortality. Conclusions: Implementation of AS and DS intervention through a pro-active ID consultation may reduce the risk of 28-day mortality of COVID-19 patients with MDROs infections.

## 1. Introduction

The SARS-CoV-2 pandemic required a significant rearrangement of healthcare systems to maintain continuity of care and protect health personnel from COVID-19. The number of critically ill patients requiring hospitalization increased significantly, causing hospital overload. Consequently, the reduced observation of infection control protocols and the high selective pressure caused by antibiotic overuse resulted in a further increase in the incidence of infections and colonization by multidrug-resistant organisms (MDROs) [1,2].

Particularly, an increase in diffusion of carbapenem-resistant *Enterobacteriaceae* (CRE) and carbapenem-resistant *Acinetobacter baumannii* and *Pseudomonas aeruginosa* occurred in intensive care units (ICUs) and medical wards, including Respiratory Diseases, Infectious Diseases, and Internal Medicine [3,4].

Of note, secondary infections caused by MDROs in the course of COVID-19 may be particularly severe, reaching an estimated overall mortality rate ranging from 30 to 53% [5,6], due to the presence of additional risk factors of mortality, including the wide administration of steroids and immunosuppressants, prolonged duration of hospitalization, central venous catheters use, and frequent need of invasive and non-invasive ventilation [6]. In addition, multidrug resistance poses a serious challenge due to the reduced available treatment options. In fact, although new drugs have been recently commercialized for “difficult-to-treat” MDROs [7], data on their use and place in therapy are still limited to clinical trials or small case series [8,9,10].

In this scenario, a multidisciplinary approach, including Infectious Disease (ID) specialists, clinical microbiologists, ICU specialists, and all other figures included in the management of patients, is pivotal to correctly manage these infections and reduce the spread of MDROs. These considerations are based on previous experience conducted to explore potential management strategies of serious infections. For instance, a large retrospective analysis performed over a 10-year timespan suggested a significant impact on reducing 30-day and 1-year mortality for resistant *S. aureus* and *Enterobacteriaceae*, and 30-day mortality for polymicrobial infections [11]; however, in this study, the incidence of *P. aeruginosa*, *Acinetobacter baumannii*, and other MDROs was too limited to draw conclusions. However, similar works, conducted on a series of *P. aeruginosa* bloodstream infections [12], and on ventilator-associated pneumonia due to MDROs [13], demonstrated a protective effect of ID consultation. Nevertheless, current data regarding the impact of ID consultation in reducing the risk of mortality associated with MDROs in the setting of COVID-19 patients hospitalized in the intensive care unit are limited.

Accordingly, the aim of this pre–post interventional study is to evaluate the impact of a pro-active IDs consultation on the management of an MDROs outbreak in a dedicated COVID-19 hospital which occurred during a SARS-CoV-2 pandemic wave.

## 2. Methods

### 2.1. Study Setting

**Figure 1:**
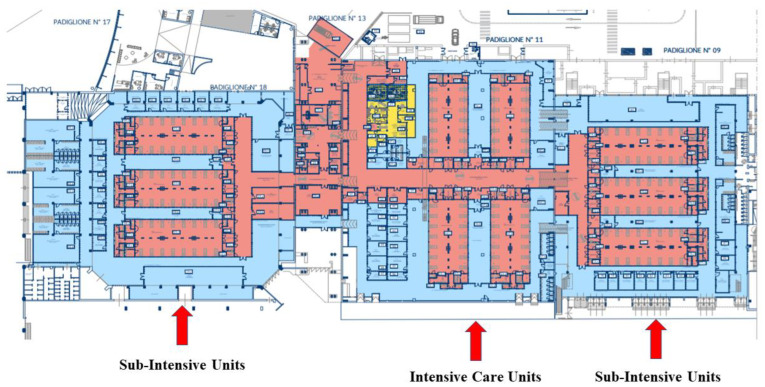
Planimetry of “Presidio Maxi-Emergenze” COVID-19 Hospital.

The study was carried out at the “Presidio Maxi-Emergenze” (PME), a dedicated COVID-19 hospital in Bari, Italy. The PME was specifically built to face the COVID-19 pandemic waves and consists of 154 beds divided between the Nephrology Unit, Respiratory Disease Unit, Internal Medicine Unit (Sub-Intensive Care Units “SICU”), and Intensive Care Units (ICU) (Figure 1). Accordingly, the PME hospitalized only patients with moderate or severe COVID-19 with age > 18 years, while subjects requiring a mild–moderate intensity of care were hospitalized at the Policlinic of Bari (in Infectious Diseases and Internal Medicine Units).

### 2.2. Study Design and Population

This is a pre–post quasi-experimental study, which was conducted from 15 March 2021 to 15 June 2021. The study is divided into a pre-phase (from 15 March to 25 April) and a post-phase (from 26 April to 15 June).

The inclusion criterium was the presence of a suspected or confirmed secondary infection in the course of COVID-19 that led to the initiation of antimicrobial therapy and/or diagnostic workup for infections.

Confirmed secondary infections present in this work were defined as follows:

- Bloodstream infections: presence of at least one blood culture positive for a Gram-negative bacteria, namely, *S. aureus*, *Enterococcus* spp., *Streptococcus* spp., or *Candida* spp.
- Pneumonia: onset of new lung infiltrate associated with a worsening of respiratory function, fever and/or other systemic sign/symptoms, and a culture from the respiratory tract positive for a Gram-negative bacteria, namely, *S. aureus* or *Aspergillus* spp.
- Urinary tract infections: fever and/or other systemic sign/symptoms (especially low urinary tract symptoms) without any other attributable cause excluding new positive urine culture for Gram-negative MDROs.

During the pre-phase, all patients were managed by the staff of the different PME Units according to the standard of care.

“Standard of care” was defined as the diagnostic and therapeutical management according only to treating physicians of PME Units, without ID consultation.

Following the onset of an outbreak of MDROs in the PME (in the pre-phase period), during the post-phase, a dedicated team of IDs consultants was involved in the management of all patients with a suspected or confirmed secondary infection.

In the post-phase, all patients enrolled in this study were multidisciplinary managed following the intervention protocol as described below.

### 2.3. Intervention

The intervention consisted of four steps:

(1) Evaluation of the extent of the problem

To verify the spreading of MDROs, a census of all colonized and/or infected patients was carried out.

Every isolate of MDROs or other pathogens at high risk of nosocomial outbreak was recorded, including the following: *Enterobacteriaceae* (*Klebsiella* spp., *E. coli*, and *Proteus* spp.) if producing carbapenemases (KPC, NDM, VIM, IMP, and OXA); carbapenem-resistant *P. aeruginosa*, carbapenem-resistant *A. baumannii*; and other AmpC-producing bacteria (*S. marcescens*, *Enterobacter* spp., *Citrobacter* spp., *Morganella morganii*, and *Providencia* spp.); and methicillin-resistant *S. aureus*, vancomycin-resistant *S. aureus*, and vancomycin-resistant *E. faecium*.

Moreover, an active surveillance of colonized patients included the following:

- Molecular rectal swab for the detection of carbapenemase genes in all patients hospitalized in PME within 72 h from the start of the intervention. In detail, the presence of the *bla* genes in the carbapenemases, including KPC and NDM, was determined on isolates from rectal swab by polymerase chain reaction (PCR) using the GeneXpert^®^ System (Cepheid). Whole genome sequencing and electrophoresis were not performed.
- Tracheal aspirate for culture test in all intubated patients.
- For patients with at least 3 episodes of diarrhea, the Clostridioides difficile test was provided in addition with stool culture.

(2) Assessment of the nosocomial infection control procedures

An audit was held by members of the ID team with the representatives of all the different wards of the PME before the post-phase starting. A review of hospital infection control procedures and protocols was carried out.

Particularly, the review regarded the following:

- Contact isolation procedures (hand washing, use of protective personal equipment, and spatial isolation) and environmental cleaning practices.
- Prevention protocols of nosocomial infections, including ventilation-associated pneumonia, catheter-associated bloodstream infections, catheter-associated urinary tract infections, and general principles of antimicrobial stewardship, with a focus on the appropriateness of prescriptions and adequate duration of therapies.

Due to the emergence condition at the time of the study, there was not sufficient time to produce internal protocols. Accordingly, we based our approach on previously published protocols, as listed below:

1. Prevention of CVC-related bloodstream infections [14];
2. Prevention of catheter-associated urinary tract infections [15];
3. Prevention of hospital-acquired pneumonia [16];
4. Antimicrobial Stewardship [17].

- A specific discussion on the importance of appropriate treatment strategies of MDROs, including carbapenem-resistant *A. baumannii* (CRAB), carbapenem-resistant *Klebsiella pneumoniae* (CR-Kp), and carbapenem-resistant *P. aeruginosa* (CR-Pa), was had.

(3) Targeted diagnostic–therapeutic interventions

The following interventions were used in the post-phase:

- The screening of all patients for carbapenemase genes on a rectal swab at the admission, once a week during the hospital stay, and at discharge (if not performed in the previous 72 h). In contrast, rectal swabbing was not used for an already-known MDR-pathogen-colonized patient; they were considered colonized and managed with contact isolation procedures until the end of hospitalization.
- The following of empirical antibiotic therapy protocols for different sites of infection (pneumonia, central-line-associated BSI, and urinary tract) based on current international guidelines; protocol deviations were recorded.
- Identification of a reference person for the implementation of prevention protocols for each unit.
- The reporting of the number of colonizations and infections weekly.

(4) Pro-active bedside evaluation of the patients

In the post-phase, the ID team performed a pro-active bedside evaluation of all hospitalized patients with suspected/confirmed secondary infections every 48–72 h, suggesting further microbiological diagnostic investigations and revising antibiotic therapy changes according to clinical and laboratory evidence. The eventual prescription of inappropriate antibiotic therapies was discussed with the unit staff and recorded. Moreover, ID consultants were available for telephone consultations if required.

General principles for inappropriate antibiotics prescription were the following:

- Antibiotics initiated without a clear suspicion/diagnosis of bacterial infection.
- Antibiotics with documented inefficacy against the causative pathogen(s) for the index infection.
- Antibiotics active against a wider spectrum of pathogen(s) than the one(s) isolated in the site of infection.
- Antibiotics prescribed against an infection which occurred in a site where they poorly penetrate.
- Antibiotics prescribed for a longer duration than what is needed to cure the infection.
- Antibiotics prescribed in combination with other molecules with redundant spectra of activity.

### 2.4. Endpoints

The primary endpoint was to evaluate the 28-day mortality rate in patients with a suspected/confirmed secondary infection.

Secondary endpoints included the analysis of the duration and costs of antimicrobial therapies.

### 2.5. Data Collection

Data about demographic aspects, comorbidities, and severity of COVID-19 were recorded.

Information about the bacterial infection and antibiotic therapies was also recorded; in particular, data regarding antibiograms, the site of the infection, severity of the infection, empirical and targeted therapies, and duration of therapies were collected. If a patient had two different suspected/confirmed infections both in the pre-phase and in the post-phase, he or she was analyzed twice.

### 2.6. Statistical Analyses

Baseline characteristics of the study population were explored through descriptive statistics including the median and interquartile range (q1–q3) for continuous variables and numbers and percentages for categorical ones; subsequently, the distributions of these variables were compared using univariable parametric or non-parametric tests, as well as the Kruskal–Wallis or Mann–Whitney U Test (where appropriate) for continuous variables, and Pearson’s χ2 test (Fisher’s exact test where appropriate) for categorical variables, according to the data distribution.

Analysis of antimicrobial costs was obtained by the following mathematical process for each day of therapy:

[(price of each antimicrobial vial × number of vials infused per day) + (price of intravenous infusion sets and all other accessories* used for one day of therapy) + cost of therapeutic drug monitoring tests, when performed (i.e., for vancomycin, teicoplanin, aminoglycosides)].

* Infusion accessories: needles, gloves, antiseptic, gauze, and plasters.

Then, to assess the predictors of 28-day mortality, a univariate Cox regression model was produced; a stepwise multivariable Cox regression was then applied to control for potential confounders and was adjusted for variables associated (*p* value < 0.1) with the endpoint at univariable analysis.

To address the non-randomized pre- or post-phase assignment, a non-parsimonious multivariable gradient boosted logistic regression model was generated to estimate, for each patient, the propensity score (PS) of being enrolled in the post-phase. Covariates included to generate the PS were determined referring to all potential risk factors for death: age, sex, severity of COVID-19, ICU stay, Charlson comorbidity index, type of infection (BSI, VAP, or others), invasive mechanical ventilation, and presence of polymicrobial infection.

A PS weighting using IPTW was then performed to create a pseudo-population with balanced covariates. Standardized differences were used to compare the balance in baseline covariates between treated and control subjects before and after weighing by the inverse probability of treatment, as appropriate [18]. Cox regression analysis was performed on the weighted sample to compare the outcome between the 2 treatment groups, and HR (95% CI) was calculated. Finally, both unadjusted and IPTW-weighted Kaplan–Meier curves of 30-day survival according to the treatment assignment were estimated. Non-parametric (log-rank or Cox) tests were used to compare survival functions in the two groups.

In all cases, a *p* value < 0.05 was considered statistically significant. Statistical analysis was performed using STATA “Special Edition” version 16.1 (STATA Corp., Lakeway Drive, TX 77845, USA).

## 3. Results

### 3.1. General Characteristics of the Study Population

In the study period, a total of 112 patients with a suspected/confirmed secondary infection were included and analyzed. A total of 89 patients were enrolled in the pre-phase, while 45 patients in the post-phase. Twenty-two of the forty-five patients were analyzed twice due to the presence of different episodes of suspected/confirmed infections.

Characteristics of the patients are summarized in Table 1. Patients in the pre- and post-phases were then compared in order to identify differences at baseline.

The population was predominantly composed of males (67% vs. 71%, *p* = 0.663), with a median age of 66 years (in both groups, *p* = 0.787) and a median Charlson comorbidity index of five (in both groups, *p* = 0.915). The rate of patients hospitalized in ICU was 70% (72% vs. 67%, *p* = 0.531), although a higher incidence of patients requiring intubation was noticed in the post-phase, despite being non-statistically significant (56% vs. 39%, *p* = 0.074).

Finally, by analyzing the infectious condition at the time of enrollment, a significantly higher incidence of colonization (31% vs. 11%, *p* < 0.001) and secondary infections (60% vs. 30%, *p* < 0.001) was noticed in the post-phase, but this was expected due to the concurrent outbreak of MDROs.

All-cause 28-day mortality was 23% (31 subjects).

Of note, the only significant difference between the two groups was a higher incidence of confirmed secondary infections (60% vs. 30%, respectively, *p* < 0.001) and colonization by at least one MDRO (31% vs. 11%, respectively, *p* < 0.001) among patients enrolled in the post-phase compared with the pre-phase.

### 3.2. Infections and Antimicrobial Therapies

Secondary infections presentation and prescribed antibiotic therapies are summarized in Table 2.

Overall, the recorded principal secondary infections were bloodstream infections (68% of cases) and hospital-acquired or ventilator-associated pneumonia (19%), while urinary tract infections were the least frequent (13%). No significant variation was observed between the two phases.

Conversely, in the post-phase, a higher number of polymicrobial/multiple infections was observed (44% vs. 12%). Similarly, etiological agents also resulted in differences: invasive fungal infections were identified only in the post-phase (10% vs. 0%). Importantly, three cases of *Candida* spp. bloodstream infections and two invasive pulmonary aspergilloses were identified in patients enrolled and treated with caspofungin and liposomal amphotericin b.

Of note, a non-significant reduction of CRAB infections was noticed (28% vs. 58%, *p* = 0.282), while a higher occurrence of KPC-Kp was recorded (33% vs. 4%, *p* = 0.007).

At the analysis of the empirical antibiotics used, the introduction of empirical antibiotic therapy protocols led to a statistically significant reduction in the use of colistin (71% vs. 30% *p* = 0.002); however, no significant difference in the use of other classes was noticed.

### 3.3. Description of Pro-Active IDs Consultation

Management of MDROs infections was based on the application of antimicrobial stewardship (AS) and diagnostic stewardship (DS) through ID consultations.

Table 3 shows the AS and DS interventions.

Notably, in our series, the predominant AS intervention was the discontinuation of antibiotic therapies (64%). In other cases, the IDs intervention consisted in a de-escalation to narrow-spectrum antibiotics (targeted therapy) or in a de-escalation to less toxic drugs (in 24% and 20% of cases, respectively).

Finally, in 33% of consultations, an optimization of antibiotic therapy was obtained, including both dosage adjustment and/or therapy escalation (defined as a switch to a targeted combination therapy or to a different drug with a wider spectrum of activity).

On the other hand, the DS intervention included in 82% of cases the request of at least one additional microbiological investigation (blood cultures, cultures of tracheal aspirate, etc.), while further instrumental investigations (echocardiography, computed tomography, etc.) were requested only in 16% of consultations.

### 3.4. Comparison of Outcomes between Pre- and Post-Phase

Interestingly, in the post-phase there was a significant reduction of the 28-day mortality (11% vs. 29%, *p* = 0.019) and of the median (q1–q3) duration of antimicrobial therapies [9 (7–10) vs. 12 (10–16) days, *p* = 0.012]. Conversely, by analyzing the median costs of the full antibiotic treatment per patient in the post- vs. pre-phase [4039 (2219.23–6628.17) vs. 6019.98 (2764.46–8962.62) euros, respectively; *p* = 0.190], the difference was non-significant.

Finally, a univariate and stepwise multivariate Cox regression analysis (Table 4) was performed to assess the risk of 28-day mortality, after adjusting for different covariates: age, sex, COVID-19 severity, infection source, CRAB or KPC-Kp as etiological agents, and post-phase attendance. While age resulted in being independently associated with an increased risk of mortality [aHR: 1.08, 95%CI: 1.03–1.13, *p* < 0.001], attendance in the post-phase proved to be a protective factor [aHR: 0.31, 95%CI: 0.10–0.92, *p* = 0.035].

Considering the observational nature of this work and the potential effect of non-randomized pre- or post-phase assignment, we made an inverse probability of treatment weighting (IPTW) adjusted model using a propensity score, yielding two well-balanced groups with no basal differences between the groups. The pseudo-cohort was matched for age, sex, severity of COVID-19, ICU stay, Charlson comorbidity index, type of infection (BSI, VAP, or others), invasive mechanical ventilation, and presence of polymicrobial infection.

Consequently, the Cox regression model was newly made on this balanced pseudo-population, confirming the increased risk of mortality associated with age (IPTW-adjusted HR = 1.05, 95%CI = 1.00–1.12) and the protective effect of post-phase attendance (IPTW-adjusted HR = 0.22, 95%CI = 0.05–0.92).

**Figure 2:**
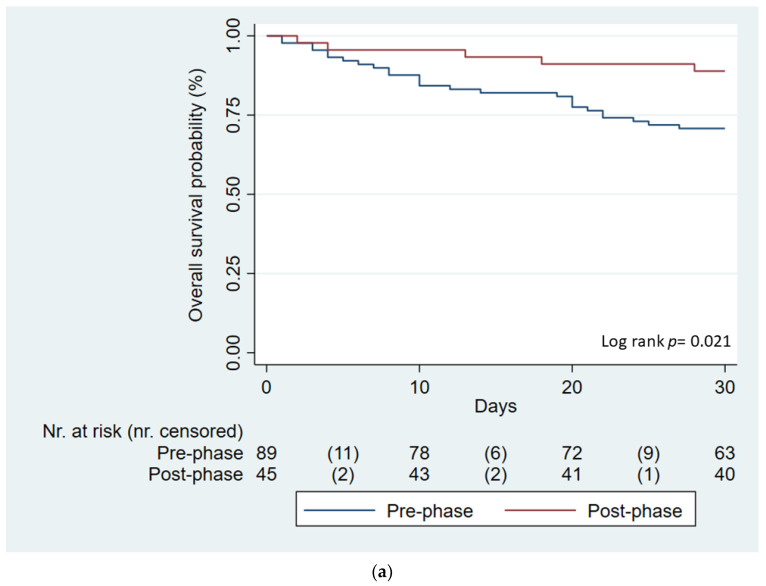

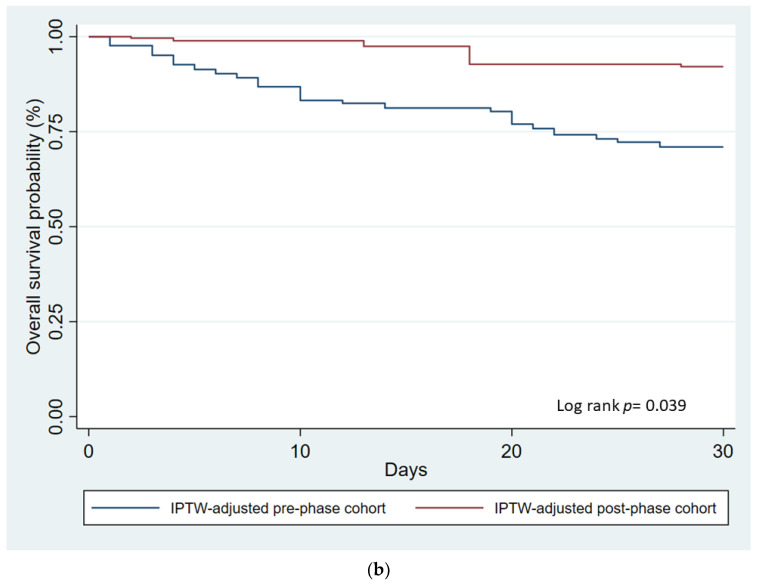
Kaplan-Meier survival curves of survival probability according to phase of enrolment in “real life” cohort (panel (**a**)) and in the and IPTW-adjusted pseudo-population (panel (**b**)). Legend: overall survival probability of patients enrolled in pre-phase (blue line) and post-phase (red line). Overall survival probability of patients enrolled in the IPTW-adjusted pre-phase population (blue line) and IPTW-adjusted post-phase population (red line).

These results were also confirmed by Kaplan–Meier curves conducted both on “real” cohort of patients (Log rank *p* = 0.021, Figure 2a) and the IPTW-adjusted pseudo-population (Log rank *p* = 0.039, Figure 2b).

## 4. Discussion

To the best of our knowledge, this is the first study evaluating the impact of a pro-active ID intervention on the outcome of secondary infections in patients with severe COVID-19, especially in the course of a hospital outbreak of MDROs.

The intervention was based on three pillars: a preliminary multidisciplinary audit aiming to implement infection prevention and control procedures, the sharing of empirical antibiotic therapies protocols, and, finally, the fulfilment of antibiotic/diagnostic stewardship principles through a pro-active bedside evaluation of ID specialists. This model was effective in reducing 28-day mortality by improving not only the approach to microbiological workup and antimicrobial therapy, but also the compliance to antibiotic stewardship principles. Importantly, in this work we predominantly focus our attention on carbapenem-resistant Gram-negative bacteria, since our intervention was requested due to the spread of Gram-negative MDROs in the Presidio Maxi Emergenze. Accordingly, we focused the activity and the analysis on reducing the incidence and mortality of these infections. Nevertheless, it is noticeable that the incidence of infections by other pathogens (including *E. coli*, *S. aureus,* and *Streptococci*) was quite low, probably due to the concurrent spread of MDROs and selective pressure caused by antimicrobials; therefore, our results are not generalizable to other kinds of infection.

Anyway, the important benefit of our intervention was surprising. Probably, one of the main reasons of this effect can be found in the high incidence of mortality in COVID-19 ICU patients with a serious MDROs secondary infection. In this critical setting, any multidisciplinary approach could represent a very valuable improvement.

However, as this is not a randomized clinical trial, we are aware that several unmeasured bias could influence mortality. Anyway, it is also true that our work was conducted in a relatively small timespan, in the context of an ICU-dedicated COVID-19 hospital, and the patients enrolled were quite comparable. On the contrary, infection incidence was significantly different between the two groups, but this was the effect of our activity in terms of infection control and antibiotic stewardship. Probably, this is also an important result.

Consequently, we conducted a propensity score adjusted analysis in order to confirm results on a pseudo-population with balanced baseline covariates. Importantly, positive effects of the intervention were confirmed.

Previous studies have already showed that ID consultation is associated with improved outcomes, especially in the case of the *Staphylococcus aureus* bacteremia and candidemia [19,20], but also in case of bloodstream infections caused by Gram-negative bacteria [21,22,23], or infections occurring in immunocompromised hosts [24,25].

However, some important epidemiological aspects of our population should be noted, which are different from previous studies. At first, all patients included in our cohort were critically ill, since they were affected by moderate/severe COVID-19 requiring high-flow oxygen therapy or non-invasive/invasive ventilation; second, several patients were affected by “difficult-to-treat” pathogens, including CRAB and KPC-Kp, or suffered from multiple episodes of infections during hospitalization. Finally, secondary infections occurring during the study period were life-threatening in most cases, including a high incidence of bloodstream infections (more than half of subjects). This highly complex setting represented a major challenge in recent COVID-19 pandemic waves, and probably was one of the major drivers of MDROs spreading [1,2].

As already highlighted, the main intervention was based on a direct bedside involvement of ID specialists in the management of patients. Similar experiences were also described by other authors in the setting of emergency departments [26] with comparable results.

Interestingly, in our experience, the discontinuation of therapies (both due to inappropriate prescription or adequate duration), the de-escalation to a targeted/narrower antibiotic spectrum, and the de-escalation to a treatment with reduced adverse events were the predominant interventions, while the escalation of antibiotic treatment or initiation of therapy was performed in only a few cases. Consequently, a significant reduction of the median duration of treatments was noticed in the post-phase. In fact, multiple studies have evidenced that a shorter duration of antibiotics is safe when guided by ID specialist evaluation [17,27].

The results of these antibiotic stewardship strategies in terms of mortality risk reduction in our population are in line with the current literature [28]: inappropriate use of antibiotics can cause antibiotic resistance up to emergency levels, multiple adverse events, further secondary infections, and in turn, deaths. Accordingly, this work supports the importance of providing stewardship interventions, also in the context of the COVID-19 pandemic [29,30].

Moreover, in this study, ID consultants suggested additional microbiological or instrumental investigations in most cases. Interestingly, this led to a significantly higher rate of fungal infections diagnosis in the post-phase compared with the pre-phase. In fact, all cases of candidemia and COVID-19-associated pulmonary aspergillosis (CAPA) were identified in the post-phase. Nevertheless, also a higher incidence of KPC-Kp infections occurred in the post-phase, but this was expected. Indeed, the outbreak of KPC-Kp was one of the reasons for the ID consultancy implementation.

Unfortunately, we failed to evidence a reduction of costs associated with antibiotic therapies. This is explainable by the high costs of drugs used for MDROs infection. Indeed, since the majority of infections was caused by “difficult-to-treat” bacteria, the use of highly expensive drugs was often unavoidable, explaining these results.

Finally, the study design was not adequate to evaluate the effect of this procedure in terms of reducing the spread of MDROs. In fact, by evaluating only patients with suspected or confirmed secondary infections, data on the incidence of colonization in other patients were lacking; similarly, data regarding environmental/surface colonization were also unavailable. Nevertheless, a trend towards a reduction of CRAB colonization was noticed, along with a significant reduction of empirical use of Colistin in the post-phase.

Despite the encouraging results observed, our study presented some limitations: at first, the small number of patients and the limited duration of the study, as well as the absence of environmental sampling to document a possible persistent source of MDROs. In fact, by performing a sample size analysis to confirm the adequacy of our study population, we calculated that, by considering at least a 10% reduction of mortality associated with our intervention, a minimum of 139 subjects was necessary to have a confidence level of 95% that the real value was within ±5% of the measured value. The sample size of the work is very similar to that which was calculated, although insufficient. Unfortunately, in this study the sample size was not pre-determined, due to the design of the work.

In addition, since the study lasted only a few months, long-term effects of this intervention were not explorable.

However, the reduced time of the study led to very uniform data recording and the inclusion of a homogenous cohort, as demonstrated by the similarity of basal conditions of the involved subjects. Furthermore, it should be noticed that this intervention led to a significant workload increase for our ID Unit, that is not sustainable over time without additional resources.

In conclusion, the results suggest that multidisciplinary teams, including the involvement of ID specialists for the management of antibiotics and cases of suspected/confirmed infections, may improve the outcome of patients with COVID-19. The effectiveness of this model could lead other healthcare organizations and departments to implement collaborations with ID specialists, particularly for the management of frail patients (post-surgical patients, solid organ transplant recipients, and subjects with hematologic cancer).

## Figures and Tables

**Table 1:** General characteristics of the study population.

	Overall (n. 134)	Pre-Phase (n. 89)	Post-Phase (n. 45)	*p* Value
Age (y), median (q1–q3)	66 (58–73)	66 (57–73)	66 (61–73)	0.787
Male sex, n. (%)	92 (69)	60 (67)	32 (71)	0.663
Charlson Comorbidity Index, median (q1–q3)	5 (3–7)	5 (3–7)	5 (4–7)	0.915
Severe COVID-19 (requiring intubation), n. (%)	60 (44)	35 (39)	25 (56)	0.074
Ward of evaluation, n. (%)				
Non-Intensive Care Units	40 (30)	25 (28)	15 (33)	0.531
Intensive Care Units	94 (70)	64 (72)	30 (67)	
Infectious condition at the time of enrollment, n (%)				
No secondary infections/colonizations	56 (42)	52 (59)	4 (9)	<0.001
Colonized by MDROs	24 (18)	10 (11)	14 (31)	<0.001
With a secondary Infection (+/− Colonization)	53 (40)	26 (30)	27 (60)	<0.001
Type of MDROs Colonization(s)	(n. 67)	(n. 33)	(n.34)	
Carbapenem-resistant A.baumannii	46 (69)	26 (79)	20 (59)	0.078
KPC-Kp	33 (49)	13 (39)	20 (59)	0.112
NDM-Kp	3 (5)	1 (3)	2 (6)	0.573

Legend: y = years; q1–q3 = first–third interquartile; MDROs = multidrug-resistant organisms.

**Table 2:** Clinical characteristics of secondary infections and prescribed antimicrobials.

	Overall (n. 53)	Pre-Phase (n. 26)	Post-Phase (n. 27)	*p* Value
Source of Secondary Infection, n (%)				
Lung	10 (19)	7 (27)	3 (11)	0.225
Bloodstream	36 (68)	17 (65)	19 (70)	
Urinary Tract	7 (13)	2 (8)	5 (19)	
Monomicrobial infection, n (%)	38 (72)	23 (88)	15 (56)	0.007
Polymicrobial/multiple infections, n (%)	9 (18)	3 (12)	12 (44)	
Etiological agent(s), n (%)				
Carbapenem-resistant A. baumannii	22 (47)	14 (54)	8 (38)	0.282
KPC-Kp	8 (17)	1 (4)	7 (33)	0.007
Other GNB	9 (19)	5 (19)	4 (19)	0.987
*Enterococcus* spp.	9 (19)	5 (19)	4 (19)	0.987
CVC-related CoNS	2 (4)	2 (8)	0	0.194
Fungi *	5 (10)	0	5 (19)	0.020
Other Gram positive **	2 (4)	2 (8)	0	0.194
Empirical antibiotic regimens prescribed before IDs consultation, n (%)	n. 60	n.24	n.36	
Carbapenems	48 (79)	21 (87)	27 (73)	0.176
Beta lactams	11 (18)	3 (12)	8 (22)	0.365
Glycopeptides	8 (13)	2 (8)	6 (16)	0.373
Lipopeptides	12 (20)	6 (25)	6 (16)	0.399
Colistin	28 (46)	17 (71)	11 (30)	0.002
Fosfomycin	7 (11)	3 (12)	4 (11)	0.840
Linezolid	10 (16)	2 (8)	8 (22)	0.171
Antifungal Agents	10 (17)	3 (12)	7 (19)	0.508
Other Agents	5 (8)	1 (4)	4 (11)	0.379

Legend: d = days; q1–q3 = first–third interquartile; Kp = *Klebsiella pneumoniae*; GNB = Gram-negative bacteria; CVC = central venous catheter; CoNS = coagulase negative *Staphylococci*. * = 3 invasive candidiasis and 2 pulmonary aspergillosis. ** = 1 Methicillin-Resistant *Staphylococcus aureus* and 1 *Streptococcus parasanguinis*.

**Table 3:** Antimicrobial and diagnostic stewardship intervention.

Pro-Active IDs Team Interventions, n (%) *	
Antimicrobial Stewardship	
Initiation/improvement of antibiotic therapy	15 (33)
De-escalation to narrow spectrum	11 (24)
De-escalation to less toxic drugs	9 (20)
Discontinuation of antibiotics	29 (64)
Diagnostic Stewardship	
Request of Microbiologic Tests	37 (82)
Request of Instrumental Tests	7 (16)

Legend: IDs = infectious diseases. * = Each percentage was calculated over the total of patients (n = 45), since for some patients, multiple interventions were performed.

**Table 4:** Univariate and multivariate Cox regression model for 28-day risk of mortality.

	Univariate Analysis			Multivariate Analysis		
	HR	95%CI	*p* Value	aHR	95%CI	*p* Value
Age, per 1 year increase	1.07	1.03–1.11	<0.001	1.08	1.03–1.13	<0.001
Male sex	1.15	0.53–2.51	0.709	1.20	0.51–2.81	0.663
Severe COVID-19 (requiring intubation)	1.20	0.59–2.43	0.604	1.50	0.61–3.65	0.371
Ward of evaluation						
Non-Intensive Care Units	1			\		
Intensive Care Units	0.64	0.31–1.32	0.235	\		
Colonization by Carbapenem-resistant A.baumannii	0.63	0.28–1.41	0.263	\		
Colonization by KPC-Kp	1.04	0.46–2.34	0.907	\		
Source of Infection						
Colonization only	1			1		
Bloodstream	1.33	0.61–2.92	0.465	1.45	0.49–4.28	0.492
Others (lung, urinary tract)	1.08	0.36–3.23	0.880	0.81	0.19–3.43	0.781
Type of Infection						
Monomicrobial infection	1			\		
Polymicrobial/multiple infections	1.53	0.41–5.66	0.522	\		
Etiological agent(s), n (%)						
Carbapenem-resistant A.baumannii	1.96	0.88–4.40	0.099	1.65	0.48–5.65	0.424
KPC-Kp	0.51	0.07–3.76	0.512	0.49	0.05–4.33	0.526
Other GNB	0.87	0.20–3.66	0.855	\		
Enterococcus spp.	0.41	0.05–3.03	0.385	\		
CVC-related CoNS	2.18	0.29–16.04	0.442	\		
Fungi	2.52	0.60–10.61	0.206	\		
Attendance in the post-phase	0.34	0.13–0.89	0.029	0.31	0.10–0.92	0.035

Legend: HR = hazard ratio; aHR = adjusted hazard ratio; d = days; Kp = *Klebsiella pneumoniae*; GNB = Gram-negative bacteria; CVC = central venous catheter; CoNS = coagulase negative *Staphylococci*.

## Data Availability

Data are available from the corresponding author upon reasonable request.
